# Effects of adiponectin on breast cancer cell growth and signaling

**DOI:** 10.1038/sj.bjc.6604166

**Published:** 2008-01-08

**Authors:** M E Grossmann, K J Nkhata, N K Mizuno, A Ray, M P Cleary

**Affiliations:** 1 Hormel Institute, University of Minnesota, 801 16th Avenue NE, Austin, MN 55912, USA

**Keywords:** adiponectin, breast cancer, proliferation, oestrogen receptor, signalling, apoptosis

## Abstract

Obesity is a risk factor for postmenopausal breast cancer. Adiponectin/Acrp30 is lower in obese individuals and may be negatively regulating breast cancer growth. Here we determined that five breast cancer cell lines, MDA-MB-231, MDA-MB-361, MCF-7, T47D, and SK-BR-3, expressed one or both of the Acrp30 receptors. In addition, we found that the addition of Acrp30 to MCF-7, T47D, and SK-BR-3 cell lines inhibited growth. Oestrogen receptor (ER) positive MCF-7 and T47D cells were inhibited at lower Acrp30 concentrations than ER-negative SK-BR-3 cells. Growth inhibition may be related to apoptosis since PARP cleavage was increased by Acrp30 in the ER-positive cell lines. To investigate the role of ER in the response of breast cancer cells to Acrp30, we established the MDA-ER*α*7 cell line by insertion of ER-*α* into ER-*α*-negative MDA-MB-231 cells. This line readily formed tumours in athymic mice and was responsive to oestradiol *in vivo*. *In vitro*, MDA-ER*α*7 cells were growth inhibited by globular Acrp30 while the parental cells were not. This inhibition appeared to be due to blockage of JNK2 signalling. These results provide information on how obesity may influence breast cancer cell proliferation and establish a new model to examine interactions between ER and Acrp30.

Obesity plays an important role in several aspects of postmenopausal breast cancer. For example, obesity is associated with increased risk for postmenopausal breast cancer (Trentham-Dietz *et al*, 2000; Morimoto *et al*, 2002; Carmichael, 2006). In addition, there are clinical studies indicating that increased BMI is associated with more aggressive breast cancer and a reduced survival period (Cleary and Maihle, 1997; Sweeney *et al*, 2004; Loi *et al*, 2005; Porter *et al*, 2006). Obesity may mediate its effects on breast cancer due to the fact that adipose tissue secretes a number of different factors that are commonly referred to as ‘adipokines’ (Vona-Davis and Rose, 2007). One adipokine potentially involved in the interaction between breast cancer and obesity is adiponectin, also known as adipocyte complement-related protein of 30 kDa (Acrp30) (Scherer *et al*, 1995). In contrast to most adipose-secreted proteins, Acrp30 is negatively correlated with body weight, body mass index, body fat, and serum leptin in women independent of age (Ryan *et al*, 2003). It has also been shown to be negatively regulated by 17β-oestradiol *in vitro* (Kim *et al*, 2005); and *in vivo*, (Im *et al*, 2006; Laughlin *et al*, 2007) although some studies do not find such regulation (Sieminska *et al*, 2005; Kleiblova *et al*, 2006).

Acrp30 was previously implicated in a number of pathological conditions. For example, decreased plasma Acrp30 levels are associated with certain insulin resistant states such as type 2 diabetes and coronary artery disease (Kadowaki and Yamauchi, 2005). Furthermore, specific factors associated with protection for some disease states such as decreased levels of triglycerides, increased insulin sensitivity, and increased high-density lipoprotein cholesterol levels are associated with increasing levels of plasma Acrp30 (Yamauchi *et al*, 2003b; Oh *et al*, 2007) Additionally general anti-inflammatory and anti-vascular effects are associated with high Acrp30 levels (Hopkins *et al*, 2007; Ouchi and Walsh, 2007). As has previously been shown for other adipokines, Acrp30 appears to have global effects on a number of different aspects of physiology and cell growth.

Acrp30 is found at high concentrations (2–20 *μ*g ml^−1^) in human serum (Arita *et al*, 1999; Bondanelli *et al*, 2001; Miyoshi *et al*, 2003; Mantzoros *et al*, 2004; Chen *et al*, 2006; Korner *et al*, 2007; Takahata *et al*, 2007). There are several different forms of Acrp30 in plasma that bind with varying affinity to the two different Acrp30 receptors, AdipoR1, and AdipoR2 (Yamauchi *et al*, 2003a). Full-length Acrp30 exists either as a trimer known as the low molecular weight form or as two larger multimers classified as middle molecular weight and high molecular weight forms (Tsao *et al*, 2002). Full-length Acrp30 binds with highest affinity to AdipoR2, which is most abundant in liver (Yamauchi *et al*, 2003a). Acrp30 can also be cleaved and exist in serum as a trimer of the smaller molecules known as globular Acrp30 (gAcrp30) (Fruebis *et al*, 2001). The gAcrp30 binds with highest affinity to AdipoR1, which has previously been shown to be expressed by a number of tissues including, brain, heart, kidney, liver, lung, spleen, and skeletal muscle (Yamauchi *et al*, 2003a). A problem that has arisen is that most publications do not specify the form(s) of Acrp30 that they are investigating. Thus, the functions of the different forms of Acrp30 and which form(s) are of the most importance in pathophysiological functions are still under active investigation.

With respect to breast cancer, lower serum Acrp30 levels have been reported for postmenopausal women with breast cancer (Bondanelli *et al*, 2001; Miyoshi *et al*, 2003; Mantzoros *et al*, 2004). *In vitro* assays have indicated that the addition of Acrp30 to the human MDA-MB-231 breast cancer cell line-induced growth arrest and promoted apoptosis (Kang *et al*, 2005; Wang *et al*, 2006). Addition of Acrp30 also blocked phosphorylation of Akt and reduced the expression of cyclin D1 (Wang *et al*, 2006). It has also been reported that under some conditions the normal response of T47D human breast cancer cells to the growth factors in serum can be blocked by Acrp30. In addition, Acrp30 was reported to have some effect on growth inhibition of two other cell lines, Hs578T and SK-BR-3, but no effect on the growth of MCF-7 and HCC-38 cells (Kang *et al*, 2005). However, other publications found that growth of MCF-7 cells was negatively regulated by Acrp30 (Dieudonne *et al*, 2006; Arditi *et al*, 2007) and one of these studies indicated that apoptosis was induced (Dieudonne *et al*, 2006). Acrp30 inhibited angiogenesis using *in vitro* and *in vivo* assays of non-breast cancer cell lines and the intratumoral injection of Acrp30 suppressed T241 fibrosarcoma tumour growth (Brakenhielm *et al*, 2004).

When discussing obesity and postmenopausal breast cancer it is important to consider that adipose tissue is the major source of oestrogen. Oestrogen is an important biological factor promoting breast cancer development in oestrogen receptor (ER) positive tumours (Hemsell *et al*, 1974). The effects of estrogens are mediated by oestrogen receptor *α* (ER*α*) and oestrogen receptor *β* (Speirs and Walker, 2007) and it is possible for the receptors to function in the absence of ligand under certain conditions (Speirs and Walker, 2007). In general, ER*α* is felt to be responsible for cell growth while ER*β* may block cell growth. Since many tissues express both genes it is likely that the ratio of the two receptors is responsible for the overall effects of oestrogen on a given tissue.

Here we have investigated the effects of Acrp30 and ER*α* in the absence of other factors such as oestradiol, leptin or serum on a number of breast cancer cell lines to clarify the role of Acrp30 on cell growth and how ER*α* without significant doses of high-affinity ligands may affect this role. In addition, we have generated new cell lines by inserting the ER*α* gene into the oestrogen unresponsive MDA-MB-231 breast cancer cell line (Cailleau *et al*, 1978) which are designated MDA-ER*α*5 and MDA-ER*α*7. We then used these lines to further investigate the potential interactions of ER*α* and different forms of Acrp30 on cell growth both *in vitro* and *in vivo*. Finally, we identified alterations in signalling pathways utilised by the ER*α*-negative and ER*α*-positive breast cancer cells in response to Acrp30 and the naturally occurring, truncated form known as gAcrp30.

## MATERIALS AND METHODS

### Cell culture

MCF-7, T47D, MDA-MB-361, SK-BR-3, and MDA-MB-231 cells were obtained from ATCC (Manassas, VA, USA) and maintained in L-15 media (ATCC, Manassas, VA, USA) with 10% fetal calf serum (FCS) (Atlanta Biologics, Lawrenceville, GA, USA) and pen/strep (Gibco, Grand Island, NY, USA). Clones of the MDA-MB-231 cell line that had been transfected with the ER*α* gene were obtained by selection with 200 *μ*g ml^−1^ of zeocin (Promega, Madison, WI, USA) and clonal lines were maintained in 200 *μ*g ml^−1^ zeocin containing media.

### Growth assays and cell signalling

**Figure 1:**
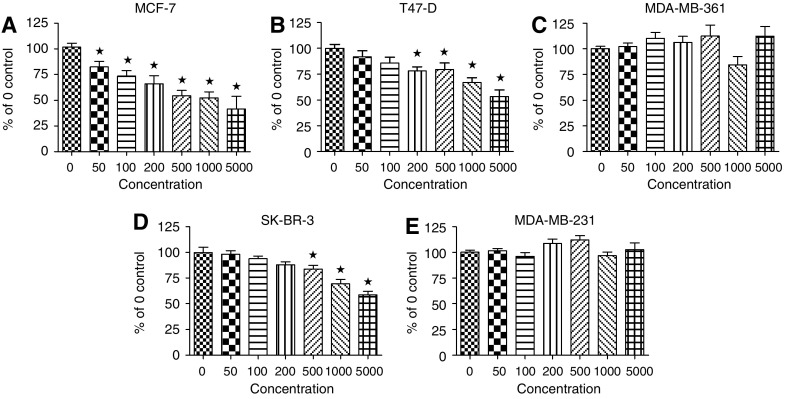
Growth curves for breast cancer cell lines in response to increasing concentrations of Acrp30. (**A**) MCF-7, (**B**) T47-D, (**C**) MDA-MB-361, (**D**) SK-BR-3 and (**E**) MDA-MB-231 cells. The concentration of Acrp30 in ng ml^−1^ is shown below each graph. Each point represents three or more wells. ^*^Indicates significantly different from 0 ng ml^−1^ as defined by ANOVA p=0.0002, Dunnett's multiple comparison post-test *P*<0.01.

**Figure 2:**
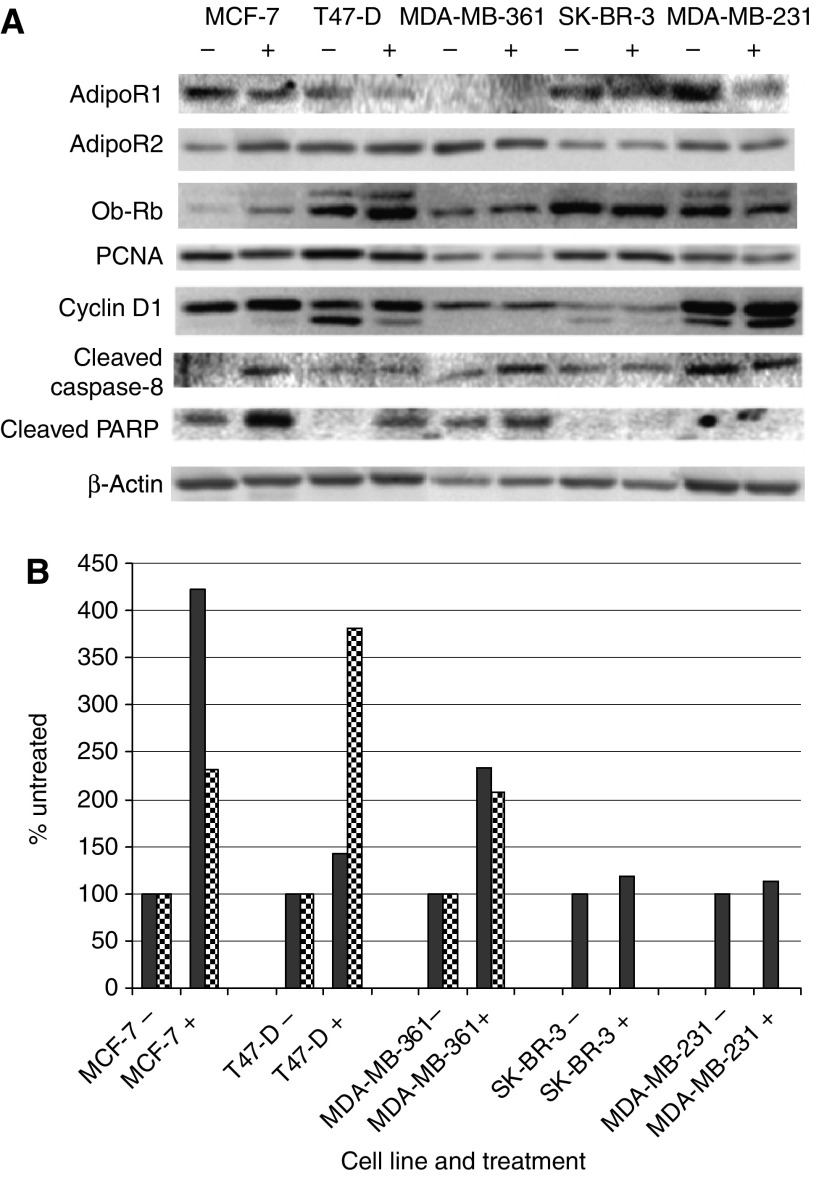
Western analysis of protein regulation by Acrp30. (**A**) The individual cell lines are shown above their two corresponding lanes on the gels. Minus symbols above the lanes indicate extracts from untreated cells and plus symbols above the lanes indicate extracts from cells treated with 1 *μ*g ml^−1^ Acrp30 for 24 h. The proteins detected are shown to the left of the figure. Abbreviations are AdipoR1=adiponectin receptor 1; AdipoR2=adiponectin receptor 2; Ob-Rb=leptin receptor isoform b; PCNA=proliferating cell nuclear antigen and PARP=Poly (ADP-ribose) polymerase. (**B**) Densitometry of cleaved caspase 8 (solid bars) and cleaved PARP (checkered bars) normalised to b-actin. The *x* axis shows the different cell lines tested without Acrp30 (−) or with Acrp30 (+). The *y* axis is percent where 100% is the value of each cell line without Acrp30. Note: cleaved PARP was not detected in SK-BR-3 and MDA-MB-231 cells under these conditions.

For the assays shown in Figure 1 on the MCF-7, T47D, MDA-MB-361, SK-BR-3, and MDA-MB-231, cells were harvested and counted using a coulter counter, plated at a density of 5 × 10^3^ cells per well in 96-well plates, and allowed to attach overnight in an incubator. The following day, complete medium was removed from the wells, replaced with serum-free medium, and the cells returned to the incubator for 18–24 h (to allow for cell cycle synchronisation). Treatment with Acrp30 was then performed and the cells incubated for 48 h, at which time a cell proliferation assay was performed. For growth assays shown in Figure 4 comparing the MDA-wt and MDA-ER*α*7 cells 5 × 10^3^ cells per well were placed in 96-well plates. The following morning the media was replaced with serum-free L-15 with or without other factors as described in the figure legend. For the experiments in Figures 2 and 5, cells were plated at 5 × 10^5^ in 6-well plates. The following day the media was replaced with various amounts of Acrp30 (RND Systems, Minneapolis, MN, USA) or gAcrp30 (Cell Sciences, Canton, MA, USA) or as described for each experiment. After 24 h of treatment the cells were either harvested or treated for various times with FCS. The growth assay was performed using 10 *μ*l of CCK-8 reagent from the Cell Counting Kit-8 as per manufacturer's instructions (Dojindo Laboratories, Japan). In this assay a formazan dye is generated by the activity of dehydrogenases in cells that is directly proportional to the number of living cells. The plates were then incubated for 1.5 to 3 h depending on the cell line in a CO_2_ incubator, after which the plates were read on an ELISA reader at 450 nm. A standard curve was included on each plate to allow for estimation of approximate cell numbers in the treated wells, as was appropriate with negative and/or positive controls.

### Western blots

All whole cell extracts were obtained as per the manufacturer's instructions for Novagen Phosphosafe extraction reagent (Merck KGaA, Darmstadt, Germany) and the amount of protein quantitated and standardised. Western analyses were performed with antibodies from Santa Cruz Biotechnology (Santa Cruz, CA, USA) except antibodies to AdipoR1 were obtained from Chemicon (Millipore, Billerica, MA, USA), AdipoR2 from Phoenix Pharmaceuticals Inc., (Burlingame, CA, USA), and anti-rabbit secondary from Cell Signalling Inc., (Danvers, MA, USA). Approximately 20 *μ*g of protein were run in each lane. The protein was transferred to PVDF membrane and checked for uniformity using Poceau S and then blocked with PBST with 5% milk. Primary antibodies were incubated in PBST with 5% milk overnight. Blots were then washed and incubated with appropriate AP-linked secondary antibodies, washed again and incubated with ECF substrate for 30 min. Blots were then visualised with a STORM 840 (Molecular Dynamics, Sunnyvale, CA, USA). Results were quantitated using UN-SCAN-IT software and normalised to *β*-actin.

### Animal experiments

Rag1 athymic C57BL6 female mice were obtained from Jackson Laboratories (Bar Harbor, ME, USA) at 5–6 weeks of age. This mouse strain was chosen because our long-term goal is to evaluate effects of dietary obesity using these cell lines and we recently reported that these mice readily develop dietary obesity when fed a purified high-fat diet (Ray *et al*, 2007). The implantation protocol was similar to the one we published previously (Ray *et al*, 2007). In brief, mice were housed three per cage and water was provided on an *ad libitum* basis. All mice were fed a purified diet based on AIN-93M (Harlan Teklad, Madison, WI, USA) recommendations for long-term maintenance of rodents. Mice in experiment 1 (exp 1) were intact but those in experiment 2 (exp 2) were ovariectomised. At 7 (exp 1) or 10 (exp 2) weeks of age mice were implanted with 120-day 17β-oestradiol pellets or control pellets (Innovative Research of America, Sarasota, FL, USA). They were then injected on the flank (exp 1) or in the mammary fat pad (exp 2) with either 3 × 10^6^ MDA-MB-231, MDA-ER*α*5 (exp 1 only) or MDA-ER*α*7 cells mixed 1 : 1 with matrigel (BD Biosciences, Bedford, MA, USA). Mice were weighed and palpated for tumours weekly for 10 weeks. At the termination of the experiment, the mice were euthanised and tumours removed, measured, and weighed. The Hormel Institute Animal Facility is AAALAC accredited. The University of Minnesota Animal Care and Use Committee approved this study.

## RESULTS

### Effects of Acrp30 on ER-positive and ER-negative human breast cancer cell lines *in vitro*

The cell lines; MCF-7 (ER+), T47D (ER+), MDA-MB-361 (ER+), SK-BR-3 (ER-), and MDA-MB-231 (ER-) were used for our initial experiments. We determined quantitatively how Acrp30 affects cell proliferation. Experiments were conducted in serum-free media to identify alterations due to direct Acrp30 signalling. Figure 1 illustrates that in the absence of serum even low levels of full-length Acrp30 are able to cause a reduction in cell proliferation of the ER*α*+ cell lines, MCF-7, and T47D (50 ng ml^−1^ or more and 200 ng ml^−1^ or more respectively). At somewhat higher concentrations (500 ng ml^−1^ or more) of Acrp30, there was also a significant reduction in growth of the ER*α*-negative SK-BR-3 cells. There was no significant reduction in the proliferative growth of MDA-MB-361 and MDA-MB-231 cell lines at these concentrations of Acrp30.

In conjunction with the cell proliferation assays, western blot analyses of a number of proteins involved in cell proliferation, apoptosis, inflammation, and vascularisation were performed to determine how their levels changed in response to Acrp30 treatment. The breast cancer cells were treated with 1 *μ*g ml^−1^ of Acrp30 for 24 h prior to cell harvest. Figure 2A highlights some of these findings showing that AdipoR2 was detected in all five cell lines. However, MDA-MB-361 cells did not have detectable AdipoR1 while the other four cell lines did. The level of AdipoR2 was upregulated in MCF-7 cells, but slightly downregulated in MDA-MB-361 and MDA-MB-231 cells 24 h after addition of 1 *μ*g ml^−1^ of Acrp30. The long form of the leptin receptor, Ob-Rb was detected in all of the cell lines and expression increased in the MCF-7 and T47D cells in response to addition of Acrp30. We measured two different proteins, which are indicators of cell growth, PCNA, and cyclin D1. We found little effect of addition of Acrp30 on either protein although it appeared that PCNA levels tended to be reduced while the cyclin D1 levels tended to increase. We also examined the opposite side of growth, death. Increased levels of cleaved caspase 8 were detected in the ER+ cell lines, MCF-7, and MDA-MB-361 but little or no increase was seen in the other cell lines in response to the addition of Acrp30. Increased PARP cleavage was detected in all of the ER+ cell lines but not in the ER− cell lines. Figure 2B shows densitometry results of the blots presented in Figure 2A for cleaved caspase 8 and cleaved PARP normalised to *β*-actin.

Tables 1, 2, 3 and 4 provide an overview of all of the different proteins tested. In addition to the results described above Table 1 shows that Acrp30 was able to regulate the receptors for the growth factors, leptin (Ob-Rb), progesterone (PR*α* and PR*β*), epidermal growth factor and androgen, in a manner that was cell type specific. However, IGFIR*α* was not regulated by Acrp30 in any of the cell lines tested. Both leptin and Acrp30 were also detected in all of the cell lines and were regulated in a cell line specific manner. In Table 2 we found that Acrp30 affected signalling through p-p38, p-JNK p-Akt (Thr308), and p-Akt (Ser473) in a cell line specific manner after 24 h of treatment. Jak2, Stat3, and PKC*α* were also regulated in a cell line specific manner. Table 3 shows a number of different proteins related to apoptosis. We found that while caspase-8 and PARP were regulated by Acrp30 (Figure 2) there was very little regulation of Bcl-2, Bcl-X_L_, Mcl-1_L_, Mcl-1_S_, Bax, and Bak although Mcl-1_S_ was increased in MDA-MB-231 cells and Bax was increased in MCF-7 cells. In addition, the levels of cleaved caspase-3, caspase-6, and caspase-9 did not change in response to Acrp30 in any of the cell lines. Table 4 shows a number of proteins involved in tumour proliferation and adhesion. We found that many of these proteins were altered by Acrp30. For example, Cox-2 expression was increased in the MCF-7 and T47D cells but not in the other lines when the cells were treated with Acrp30. Expression of VEGF was increased in the T47D and SK-BR-3 cells and decreased in the MDA-MB-361 cells when Acrp30 was added. Lamin*β*-3 was increased in MCF-7 cells and E-cadherin was increased in T47D cells but decreased in MDA-MB-361 cells. All of this suggests that Acrp30 has profound effects on many different genes involved in tumour proliferation and adhesion but in a cell type specific manner.

### Addition of ER-*α* to MDA-MB-231 ER-negative human breast cancer cells

Our results in Figure 1 suggest that the ER status of the breast cancer cells may cause them to be more sensitive to the growth inhibition of Acrp30. To better examine the role of ER*α* on the Acrp30 effects we added ER*α* into the oestrogen unresponsive cell line MDA-MB-231 (MDA-wt) using liposome-mediated transfection. Initially, twelve clonal cell lines, MDA ER*α* line 1 to MDA ER*α* line 12, were selected, tested, and found to express ER*α* protein (data not shown). We then measured cell growth in early passage clones in response to 2.5 ng ml^−1^ oestradiol after 24 h using growth assays for six of the faster growing cell lines (data not shown). We also purposely chose to test several slower growing cell lines. To do this, cells from five clones were manually counted after 5 and 7 days of 2.5 ng ml^−1^ oestradiol treatment (data not shown). Several clones with modestly higher growth rates in response to 2.5 ng ml^−1^ oestradiol compared to the original MDA-wt cell line were identified in these two initial assays. The ER*α* line 5 cells (MDA-ER*α*5) was selected as a faster growing cell line for further testing. They have a similar doubling time to the MDA-wt cells. We also chose ER*α* line 7 cells (MDA-ER*α*7) as a slower growing cell line for additional testing.

**Figure 3:**
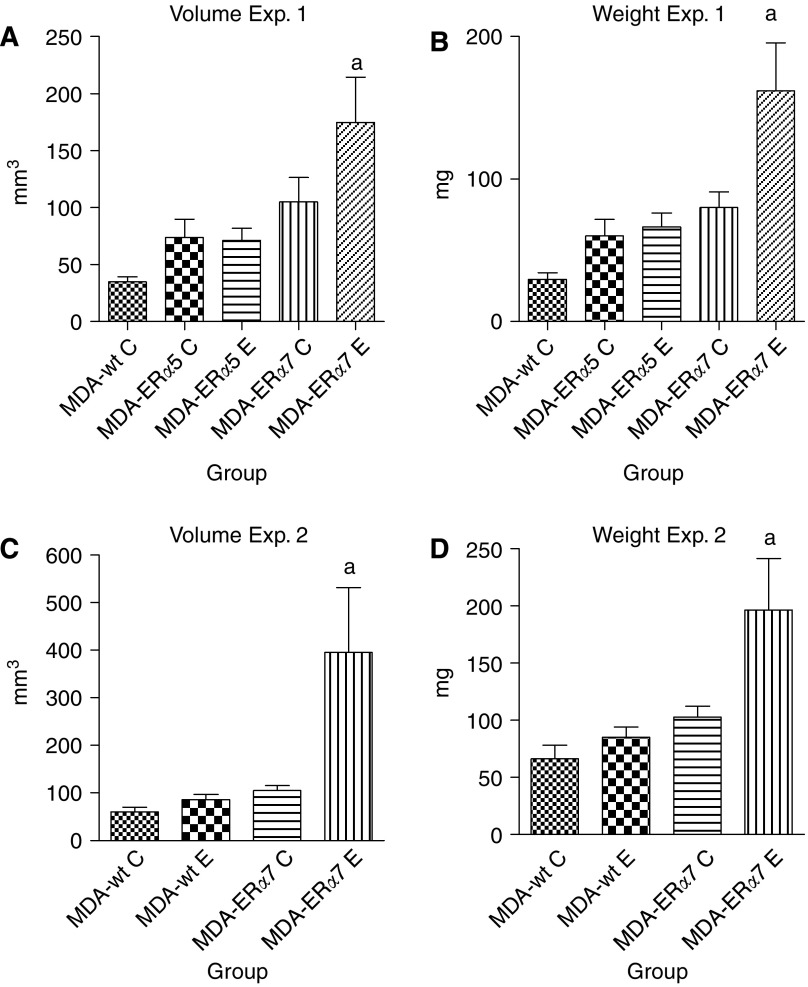
Growth of MDA-wt and clones expressing ER*α* in response to oestradiol *in vivo*. Rag1 mice were injected with the cell lines shown along the *x* axis in the presence (E) or absence (C) of oestradiol pellets. Mice were intact for experiment 1 and ovariectomised in experiment 2. Tumours were excised, weighed and measured at the end of the experiment. The average volume in millimetres of the tumours are shown in (**A**) and (**C**) and the average weight in milligrams are shown in (**B**) and (**D**) along the *y* axis for experiments one and two respectively. The. volume for each individual tumour was computed according to the formula length ((longest dimension) × width squared (widest point at right angle to length) × 0.52). The different groups are shown along the *x* axis. Bars represent standard error of the mean. An ‘a’ above the bars shows groups that are statically different than all other groups as determined by ANOVA with Newman–Keul's multiple comparison post-test (*P*<0.01).

We felt that the most relevant way to determine if the addition of ER*α* is providing a growth advantage for the MDA-ER*α* clones was to perform *in vivo* experiments. Therefore, we injected the MDA-ER*α*5, MDA-ER*α*7, and MDA-wt cells into athymic Rag1 C57BL/6 female mice using oestradiol or placebo pellets in the mice implanted with the MDA-ER*α* clones. The MDA-ER*α*7 cells in mice with exogenous oestrogen formed heavier tumours (Figure 3A) compared to the other groups (164 *vs* 24–81 mg) and grew to a considerably larger size (Figure 3B) compared to any of the other experimental groups (172 *vs* 41–103 mm^3^) (ANOVA *P*<0.01 with Newman–Keul's multiple comparison test *P*<0.01 or more for all groups). The MDA-wt grew slowest although this was not statistically significant compared to any of the other groups except for the MDA-ER*α*7 with exogenous oestrogen. The tumours in the groups inoculated with MDA-ER*α*5 cells in the presence or absence of exogenous oestradiol and the MDA-ER*α*7 cells in the absence of exogenous oestradiol all grew at almost the same rate.

To confirm this finding we performed a second experiment implanting just the MDA-wt and MDA-ER*α*7 cells. We again utilised the Rag-1 mice but this time the mice were ovariectomised. In addition, both the mice implanted with MDA-ER*α*7 cells and those implanted with MDA-wt cells received either oestradiol or placebo pellets. As before, the MDA-ER*α*7 cells implanted into the mice that received exogenous oestradiol grew much faster as measured by tumour weight (Figure 3C; 198 *vs* 68–102 mg) and size (Figure 3D; 399 *vs* 53–103 mm^3^) in a manner that was statistically significant (ANOVA *P*<0.01 with Newman–Keuls multiple comparison test *P*<0.01 or more for all groups). The MDA-wt cells in the absence of oestradiol pellets grew slightly slower than the other cells while the MDA-wt cells in the presence of oestradiol pellets and the MDA-ER*α*7 cells in the absence of oestradiol pellets grew at similar rates. However, none of these three groups were significantly different from the others as determined by ANOVA with Newman–Keul's post test. The tumour take for the MDA-ER*α*7 cells was 8 of 10 for exp 1 and 7 of 8 for exp 2 in the presence of exogenous oestradiol. In the absence of exogenous oestradiol the tumour take for the MDA-ER*α*7 cells was 10 of 10 and 11 of 11 for exp 1, and exp 2, respectively. These results establish that the MDA-ER*α*7 cell line readily forms tumours *in vivo* and is oestrogen responsive *in vivo*.

### *In vitro* growth and signalling of MDA-ER*α*7 and MDA-wt cells in response to Acrp30

**Figure 4:**
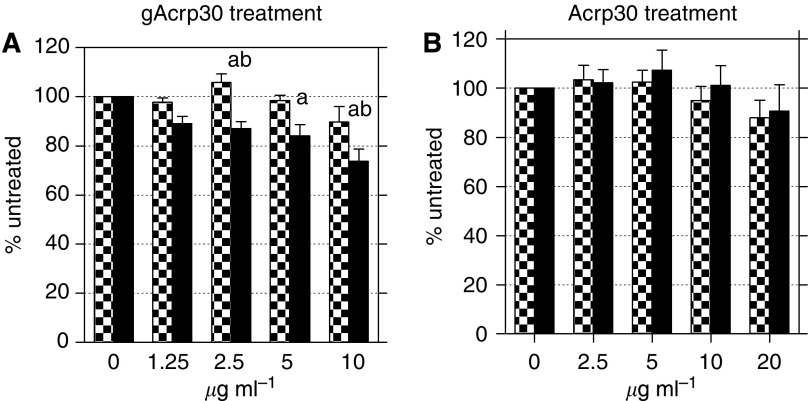
Growth of MDA-wt and MDA-ER*α*7 cells in response to gAcrp30 and Acrp30 *in vitro*. The MDA-wt (checkered bars) and MDA-ER*α*7 (filled bars) cells are shown with cell growth as a percent along the *y* axis. Cells in only serum-free L-15 were considered to be 100%. The concentrations of gAcrp30 (**A**) or Acrp30 (**B**) are shown along the *x* axis. The assays were performed in triplicate and repeated a total of three separate times. Bars represent standard error of the mean of the three different experiments. An ‘a’ corresponds to significantly different from the 0 control of the same cell line as determined by ANOVA with the Dunnett's post-test and ‘b’ corresponds to a significant difference between the two cell lines at the same concentration as determined by ANOVA with the Newman–Keul's test.

In these *in vitro* studies we increased the level of Acrp30 to a range of 2.5–20 *μ*g ml^−1^ to obtain more physiological levels and utilised only the MDA-wt and MDA-ER*α*7 cell lines to evaluate the potential interplay of ER*α* and Acrp30. We also examined the role of two different forms of Acrp30, full length (Acrp30), and globular (gAcrp30). The use of gAcrp30 resulted in a reduction in cell proliferation for the MDA-ER*α*7 cells (Figure 4A) that was statistically significant at 2.5–10 *μ*g ml^−1^ of gAcrp30 tested (ANOVA *P*=0.0001, Dunnett's *P*<0.05–0.01). There was also a slight reduction in the growth of the MDA-wt cells after 48 hours but this was not significant. The difference in growth between the MDA-ER*α*7 and MDA-wt cells was statistically significant at Acrp30 concentrations of 2.5 and 10.0 *μ*g ml^−1^ (Newman–Keuls *P*<0.05). The treatments of the two cell lines also differed from each other as a whole by the Student's *t*-test *P*=0.0254. We found only a modest decrease in cell growth by full length Acrp30 after 48 h of treatment (Figure 4B). There was a slight downward trend at the highest level of Acrp30 but it was not statistically significant. This indicates that the ER+ MDA-ER*α*7 cells are more responsive to growth inhibition by gAcrp30 than the ER-MDA-wt cells.

**Figure 5:**
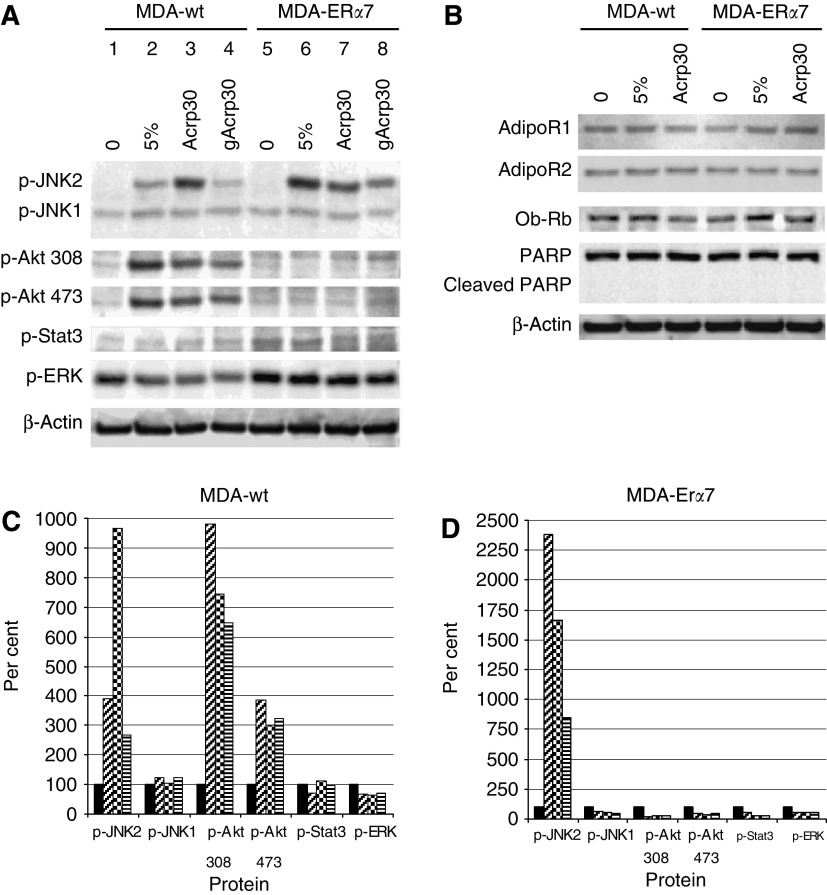
Alteration of signalling pathways by Acrp30 and gAcrp30. MDA-wt and MDA-ER*α*7 cells were treated with 10 *μ*g ml^−1^ of gAcrp30 or full-length Acrp30 for 24 h in serum-free media. After pretreatment 5% FCS was added to the cultures indicated for 15 min (**A**) or 24 h (**B**). The different antibodies used are shown along the side and the cell lines with treatments are shown above the lanes. In (**C**) (MDA-wt) and (**D**) (MDA-ER*α*7) densitometry is shown of (**A**) that has been normalised to *β*-actin. The *x* axis is the different proteins and the *y* axis is percent where the unstimulated control is set to 100%. Solid bars are untreated (lanes 1 and 5), diagonal slashes are 5% FCS (lanes 2 and 6), checkered bars are 5% FCS with Acrp30 pretreatment (lanes 3 and 7) and horizontal bars are 5% FCS with gAcrp30 pretreatment (lanes 4 and 8).

We have investigated some of the possible mechanisms for this growth reduction by identifying increases or decreases in phosphorylation of several growth-associated signalling pathway. We found that gAcrp30 and Acrp30 treatment inhibited the signalling in MDA-ER*α*7 and MDA-wt cells associated with stimulation of resting cells by the growth factors found in FCS. Figure 5A shows phosphorylation as an indicator of activation for, JNK1, JNK2, Akt Thr308, Akt Ser473, Stat3, and ERK. In the ER+ MDA-ER*α*7 cells, the phosphorylation of JNK2 but not JNK1 is greatly stimulated by 5% FCS and the increase can be inhibited by gAcrp30 and to a lesser extent Acrp30 (Figure 5A and 5D). However, in the ER-MDA-wt cells JNK2 was not stimulated as much and addition of Acrp30 actually resulted in an increase in JNK2 phosphorylation. A major difference between the two cell lines is that FCS stimulation of the MDA-wt cells results in phosphorylation of the Akt pathway. Both Thr308 and Ser473 are phosphorylated by the presence of FCS and the levels of phosphorylation of both are decreased by Acrp30 and to a greater extent by gAcrp30 (Figure 5A and 5C). The ER+ MDA-ER*α*7 cells did not show an increase in Akt phosphorylation in response to FCS. We also tested two other signalling pathways for activation in the MDA-wt and MDA-ER*α*7 cells. We did not find any changes in the phosphorylation of either ERK or Stat3. Finally, we investigated the effects of high-dose (20 *μ*g ml^−1^) Acrp30 after 24 hours of treatment on AdipoR1, AdipoR2 Ob-Rb, Ob-R, and PARP (Figure 5B). We found that while all of these proteins are expressed by both the MDA-wt and the MDA-ER*α*7 cells none of these proteins were significantly regulated in this experiment. In addition, we did not see any PARP cleavage under these conditions.

## DISCUSSION

The number of women in the United States who are obese has doubled in the past 25 years. As the average body mass index climbs, the overall levels of Acrp30 will decline making this area of research progressively more important (Smigal *et al*, 2006; Wyatt *et al*, 2006). We have been able to show that five different breast cancer cell lines express either AdipoR1 and/or AdipoR2 protein. Previously, mRNA for AdipoR2 and protein for AdipoR1 had been detected in MCF-7, T47D and MDA-MB-231 cells (Dieudonne *et al*, 2006; Arditi *et al*, 2007). We confirmed the protein expression and found that AdipoR1 protein was also expressed by SK-BR-3 cells. Our results also indicate AdipoR2 protein was expressed by MCF-7, T47D, MDA-MB-231, and MDA-MB-361 breast cancer cells. Additionally, these receptors appear to be functional since in some breast cancer cell lines growth was inhibited by the addition of Acrp30 (Figure 1). We did not find large changes in the cell cycle indicators, PCNA or cyclin D1, possibly due to the use of serum-free media, which may have caused the cells to enter a G_0_ state in which PCNA and cyclin D levels were already relatively low. We did find that caspase-8 and PARP appeared to be activated by Acrp30 in ER+ cell lines (Figure 2). These findings suggest that apoptosis was initiated in ER+ but not ER− cell lines by Acrp30 in this serum-free experiment.

To better understand why some women would be more adversely affected by obesity, we initially compared both Her2/neu status and ER*α* status in conjunction with the response of the cell lines we examined for decreased proliferation in response to Acrp30. Table 5 shows that two of the lines that did not express Her2/neu, the MCF-7 and T47D, did decrease their proliferation in response to Acrp30 but that the MDA-MB-231 line which also lacks Her2/neu did not decrease its proliferation in response to Acrp30. Of the two lines that expressed Her2/neu, the SK-BR-3 cells did have a decrease in proliferation but the MDA-MB-361 cells did not. When we examined the status of ER*α* in these cell lines as compared to decreased proliferation in response to Acrp30, we found that the MCF-7 and T47D cells which express ER*α* had a decrease in proliferation but that the MDA-MB-361 cells which also express ER*α* did not exhibit the same decrease. Of the cells that did not express ER*α*, SK-BR-3 had a decreased proliferation rate at higher Acrp30 levels as compared to MCF-7 and T47D cells but the MDA-MB-231 cells did not. Because there appeared to be a relationship between expression of ER*α* and decreased sensitivity of the cells’ antiproliferation response to Acrp30, we chose to further investigate the interaction of Acrp30 and ER*α* by developing a new cell line designated MDA-ER*α*7. This cell line expresses ER*α* and is oestrogen responsive *in vivo* since it exhibited increased growth in response to exogenous oestradiol (Figure 3). Other investigators have transfected ER*α* into MDA-MB-231 cells and have found no change in growth (Touitou *et al*, 1991; Bandyopadhyay *et al*, 2007) or even a decrease in the presence of oestradiol (Jiang *et al*, 1992). The difference may be attributable to clonal variation or the fact that we first tested the MDA-ER*α*7 cells soon after selection. Interestingly, while initial experiments with very early passage MDA-ER*α*7 cells suggested it was oestrogen responsive, *in vitro* testing with later passages of MDA-ER*α*7 cells showed little or no increase in growth in response to oestradiol (data not shown) despite the fact that similar passages were responsive to oestradiol *in vivo*. This may be due to interactions between oestradiol and other growth factors that would be possible *in vivo* but that did not occur in our *in vitro* system due to the fact that we used serum-free media to perform our oestradiol growth experiments. These results illustrate that *in vivo* growth and *in vitro* growth can be very different.

When we tested the effects of Acrp30 and gAcrp30 using *in vitro* assays we found that MDA-ER*α*7 cells have decreased cell growth and were more sensitive to gAcrp30 as compared to the parental MDA-wt cells (Figure 4A). These decreases in proliferation may be attributable to decreased signalling through JNK2 since phosphorylation of JNK2 by FCS is inhibited by adiponectin, particularly gAcrp30 (Figure 5). The concentration levels of the receptors, AdipoR1 and AdipoR2, did not seem to be involved in cellular response since they were very similar (Figure 5B). Interestingly, the ER*α*+ MDA-ER*α*7 cells did not show phosphorylation of Akt following serum stimulation even though the parental MDA-wt cells did (Figure 5).

We did not find any indication of growth inhibition in relationship to the cell cycle as no changes in levels of cyclin D1 or PCNA in the absence of serum were detected. However, we did find that the ER+ cells treated with low doses of Acrp30 appear to have an increase in apoptosis. This was suggested by the increase in cleaved caspase-8 and cleaved PARP in these cells. Other reports have found that apoptosis is involved in growth inhibition by Acrp30 by the use of TUNEL or annexin V assays (Kang *et al*, 2005; Dieudonne *et al*, 2006; Wang *et al*, 2006). We have extended those earlier observations by identifying increased cleavage of caspase-8 and PARP following treatment with Acrp30. We also looked for cleavage of caspase-3, caspase-6, and caspase-9 but did not find any changes due to Acrp30 treatment suggesting that under these conditions the parts of the apoptosis pathway controlled by these proteins was not being utilised.

Several groups reported previously that MDA-MB-231 cells were growth inhibited by Acrp30 (Kang *et al*, 2005; Wang *et al*, 2007). However, in our hands proliferation of this cell line was not statistically inhibited by the addition of Acrp30. This lack of agreement may be attributable to different culture conditions, subclone variation of the cells and/or the use of different sources of Acrp30. We purchased Acrp30 while the other groups made theirs using at least three different methods.

Our study is one of the first to investigate the function of gAcrp30 *vs* Acrp30. Very little work has been done in this area with only one previous mention that gAcrp30 did not affect MDA-MB-231 cells growth but the data were not shown (Wang *et al*, 2006). In our hands the addition of gAcrp30 to MDA-MB-231 cells resulted in fewer cells after 48 hours but the reduction was not statistically significant. Previous work has shown that AdipoR1 has a higher affinity for gAcrp30 (Yamauchi *et al*, 2003a). Our study was not able to address which of the Acrp30 receptors was most important for the reduction in cell number since our cell lines expressed both receptors. Additional work using shRNA to knockout AdipoR1, AdipoR2 or both receptors may help to clarify the roles of the two receptors with regards to cell growth and/or death.

Here, cell growth inhibition by Acrp30 was examined independently of any other serum factors. However, the interplay between Acrp30 and other growth factors has been shown to be important for Acrp30's ability to inhibit cell proliferation (Wang *et al*, 2005). With respect to breast cancer, and obesity, leptin has been an adipokine implicated in mammary tumorigenesis (Hu *et al*, 2002; Cleary *et al*, 2003; Frankenberry *et al*, 2006; Garofalo *et al*, 2006). Overweight or obese individuals usually have elevated levels of the growth factor leptin, which is positively correlated to body mass index while Acrp30 is negatively correlated with body mass index. We and others are now showing that Acrp30 may be an important negative regulator of breast cancer cell growth (Miyoshi *et al*, 2003; Kang *et al*, 2005; Dieudonne *et al*, 2006). In particular, we are trying to assess the interrelationship of these two adipokines on tumour growth and development. It is likely that a balance between the two adipokines will determine if a tumour increases or decreases in size.

Our current study has illustrated that Acrp30 may be involved in apoptosis as well as inhibition of growth factors found in serum which initiate proliferation through the JNK and Akt pathways. This is consistent with a prior study showing that pretreatment of human aortic smooth muscle cells with Acrp30 inhibited the function of growth factors in serum (Wang *et al*, 2005). Previously it had been anticipated that there would be increased levels of growth factors released during refeeding following calorie restriction which would enhance tumour development; however, our previous *in vivo* findings indicated that intermittent calorie restriction resulted in increased mammary tumour latency and decreased tumour incidence (Cleary *et al*, 2002, 2007). Human findings on calorie restriction indicate that serum Acrp30 levels increased following weight loss either through diet (Weiss *et al*, 2006), MacLean vertical banded gastroplasty or biliopancreatic diversion with duodenal switch (Kotidis *et al*, 2006). We are currently investigating the levels of Acrp30 during calorie restriction *in vivo* with ongoing mouse mammary and prostate tumorigenesis studies. We feel that factors in the serum of the mice, specifically adipokines have the ability to function as both growth stimulators such as has been shown for leptin or as growth inhibitors such as seems to be the case with Acrp30 for breast cancer. Additional experiments will confirm and define the roles of adipokines such as Acrp30 and leptin and determine how the balance of the two impact breast cancer cell growth and death.

## Figures and Tables

**Table 1:** Acrp30 regulation of potential growth factors and their receptors

	**MCF-7**	**T47D**	**MDA-MB-231**	**MDA-MB-361**	**SK-BR-3**
AdipoR1	−	−	−	ud	−
AdipoR2	+	n/c	−	−	n/c
Ob-Rb	+	+	−	n/c	n/c
PRα	+	−	ud	ud	ud
PRβ	ud	+	ud	ud	ud
EGFR	ud	n/c	−	ud	−
p-EGFR	ud	ud	−	ud	n/c
LXRα	n/c	n/c	n/c	n/c	n/c
LXRβ	+	+	n/c	n/c	n/c
AR	n/c	+	ud	−	ud
IGFIRα	n/c	n/c	n/c	n/c	n/c
Leptin	+	+	n/c	n/c	n/c
Acrp30	−	+	+	n/c	+

−=decrease; n/c=no change; +=increase; AdipoR=adiponectin receptor; AR=androgen receptor; EGFR=epidermal growth factor receptor; IGFIR=insulin-like growth factor-I receptor; LXR=liver x receptor; Ob-R=leptin receptor; p=phosphorylated; PR=progesterone receptor; ud=undetected.

**Table 2:** Acrp30 alteration of potential signalling proteins.

	**MCF-7**	**T47D**	**MDA-MB-231**	**MDA-MB-361**	**SK-BR-3**
Jak2	+	+	−	−	n/c
Stat3	n/c	+	+	n/c	−
PI3K	n/c	n/c	n/c	n/c	n/c
p-p38	−	n/c	n/c	n/c	n/c
p-JNK1/2	+	n/c	n/c	n/c	−
p-Akt308	−	+	n/c	n/c	n/c
p-Akt473	n/c	+	n/c	n/c	n/c
PKCα	+	+	n/c	−	n/c

−=decrease; n/c=no change; +=increase; Jak=janus kinase; JNK=c-jun amino-terminal kinase; PI3K=phosphatidylinositol 3-kinase; PKC=protein kinase C=Stat: signal transducer and activator of transcription; ud=undetected.

**Table 3:** Effects of Acrp30 on proteins involved in apoptosis

	**MCF-7**	**T47D**	**MDA-MB-231**	**MDA-MB-361**	**SK-BR-3**
Bcl-2	n/c	ud	n/c	n/c	n/c
Bcl-X_L_	n/c	n/c	n/c	n/c	n/c
Mcl-1_L_	n/c	n/c	n/c	n/c	n/c
Mcl-1_S_	n/c	n/c	n/c	n/c	+
Bax	+	n/c	n/c	ud	n/c
Bak	n/c	n/c	n/c	n/c	n/c
FL-Caspase-3	ud	−	+	−	+
Cleaved Caspase-3	ud	n/c	n/c	n/c	n/c
FL-Caspase-6	+	−	n/c	ud	−
Cleaved Caspase-6	ud	ud	ud	ud	ud
FL-Caspase-8	+	−	−	n/c	+
Cleaved Caspase-8	+	n/c	−	+	n/c
Caspase-9	+	−	n/c	n/c	n/c
Cleaved Caspase-9	ud	ud	ud	ud	ud
FL-PARP	n/c	+	ud	ud	ud
Cleaved PARP	+	+	ud	+	ud

−=decrease; n/c=no change; +=increase; Cl=cleaved; Fl=full length; PARP=poly-ADP-ribose polymerase; ud=undetected.

**Table 4:** Effects of Acrp30 on proteins involved in tumor proliferation and adhesion

	**MCF-7**	**T47D**	**MDA-MB-231**	**MDA-MB-361**	**SK-BR-3**
TP53	+	+	n/c	ud	n/c
p-TP53(Ser 315)	N/c	n/c	n/c	−	n/c
p-TP53(Ser392)	+	n/c	−	n/c	−
p-TP53(hSer20)	n/c	+	n/c	ud	+
SHBG	+	n/c	n/c	−	−
IGFBP3	+	n/c	n/c	n/c	n/c
Mdr	n/c	n/c	n/c	n/c	ud
PCNA	−	−	−	n/c	n/c
Cyclin D1	+	+	n/c	n/c	n/c
Cox2	+	+	n/c	n/c	n/c
VEGF	n/c	+	n/c	−	+
p-*β*-catenin	ud	n/c	n/c	ud	+
*δ*-catenin	+	−	−	n/c	ud
Laminin*β*-3	+	n/c	n/c	n/c	n/c
E-cadherin	n/c	+	n/c	−	ud
PSA	ud	ud	−	n/c	+

−=decrease; n/c=no change; +=increase; Cox-2=cyclooxygenase 2; IGFBP=IGF binding protein; Mdr=multi-drug resistance protein; PCNA=proliferating cell nuclear antigen; PSA=prostate specific antigen; SHBG=sex hormone-binding globulin; ud=undetected; VEGF=vascular endothelial growth factor.

**Table 5:** ERα and Her2/neu status of breast cancer cell lines and response to Acrp30

	**ERα-positive**	**Overexpress Her2/neu**	**Proliferation in response to Acrp30**
MCF-7	+	−	−−−
T47-D	+	−	−−
MDA-MB-361	+	++	No
SK-BR-3	−	+++	−
MDA-MB-231	−	−	No

−=decrease; +=increase.
